# Gabapentin-Associated Movement Disorders: A Literature Review

**DOI:** 10.3390/medicines10090052

**Published:** 2023-09-06

**Authors:** Jamir Pitton Rissardo, Ursula Medeiros Araujo de Matos, Ana Letícia Fornari Caprara

**Affiliations:** 1 Neurology Department, Cooper University Hospital, Camden, NJ 08103, USA; 2 Medicine Department, Federal University of Rio de Janeiro (UFRJ), Rio de Janeiro 21941-853, Brazil; ursulamatos@id.uff.br; 3 Medicine Department, Federal University of Santa Maria (UFSM), Santa Maria 97105-900, Brazil; ana.leticia.fornari@gmail.com

**Keywords:** neurontin, ci-945, goe-3450, dm-1796, movement disorder, myoclonus, dyskinesia, seizure, side effect, neuropathy

## Abstract

Background: Gabapentin (GBP)-induced movement disorders (MDs) are under-recognized adverse drug reactions. They are commonly not discussed with patients, and their sudden occurrence can lead to misdiagnosis. This literature review aims to evaluate the clinical–epidemiological profile, pathological mechanisms, and management of GBP-associated MD. Methods: Two reviewers identified and assessed relevant reports in six databases without language restriction between 1990 and 2023. Results: A total of 99 reports of 204 individuals who developed a MD associated with GBP were identified. The MDs encountered were 135 myoclonus, 22 dyskinesias, 7 dystonia, 3 akathisia, 3 stutterings, 1 myokymia, and 1 parkinsonism. The mean and median ages were 54.54 (SD: 17.79) and 57 years (age range: 10–89), respectively. Subjects were predominantly male (53.57%). The mean and median doses of GBP when the MD occurred were 1324.66 (SD: 1117.66) and 1033 mg/daily (GBP dose range: 100–9600), respectively. The mean time from GBP-onset to GBP-associated MD was 4.58 weeks (SD: 8.08). The mean recovery time after MD treatment was 4.17 days (SD: 4.87). The MD management involved GBP discontinuation. A total of 82.5% of the individuals had a full recovery in the follow-up period. Conclusions: Myoclonus (GRADE A) and dyskinesia (GRADE C) were the most common movement disorders associated with GBP.

## 1. Introduction

Gamma-aminobutyric acid (GABA) acts as the main inhibitory neurotransmitter in the central nervous system (CNS), and the under regulation of this amino acid is associated with epilepsy [1]. GABA does not cross the blood–brain barrier, so, in 1976 when Satzinger et al. originally developed gabapentin (GBP), 1-(aminomethyl) cyclohexane acetic acid, they aimed to create a lipophilic GABA analog capable of crossing the blood–brain barrier through passive diffusion [2]. GBP was not metabolized hepatically and was not bound to plasma proteins. However, years later, contrary to the original blood–brain barrier diffusion hypothesis, Su et al. discovered that GBP was actually delivered to the CNS through the system L-transporter in cultured astrocytes in a rat brain cortex [3].

Following these observations, studies with experimental models of seizures in mice tested the effectiveness of GBP [4]. Crawford et al. assessed for the first time the effectiveness of GBP in humans. The authors tested GBP in patients with focal and generalized uncontrolled epilepsy. Crawford et al. described a decrease of approximately thirty percent in the frequency of seizures, and a dose-related antiepileptic effect was observed [5]. In 1993, the US GBP Study Group performed a two-year-long multicenter, open-label study about the effectiveness and safety of GBP as an add-on therapy in patients with drug-resistant focal seizures. The percentage of adverse events was ten percent, and the most common adverse events reported were nystagmus, somnolence, diplopia, tremor, ataxia, and dizziness. In this context, 4% of the patients withdrew GBP due to adverse events [6]. In December 1993, the U.S. Food and Drug Administration (FDA) approved GBP as an adjuvant antiepileptic drug for focal seizures in adults [7]. Eventually, in 2002, GBP received U.S. FDA approval for treating post-herpetic neuralgia. With GBP on the market, several other open-label studies suggested this drug might also have utility against anxiety and panic disorders [8]. However, high GBP doses were required to manage these neuropsychiatric disorders [9].

**Figure 1:**
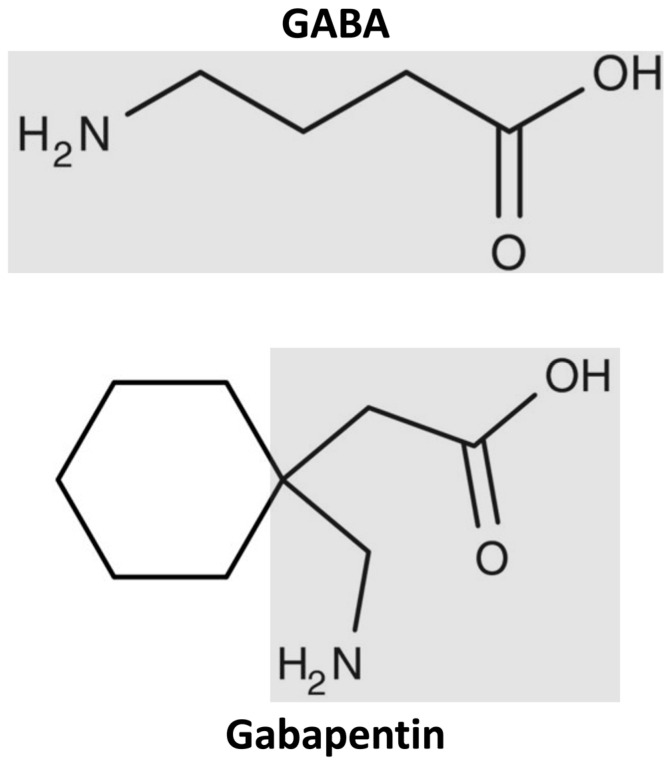
Structure of gamma-aminobutyric acid (GABA) and gabapentin.

The mechanism of action of GBP is not completely understood. Interestingly, although GBP is a GABA analog, it does not influence GABA metabolism, transport, or GABA receptors (Figure 1). Some studies have hypothesized that GBP inhibits the α2δ subunit-containing voltage-dependent calcium channels (VDCCs) involved in the postsynaptic neurotransmitter release [10]. Thus, this disruption in calcium channel trafficking would block new synapse formation. An alternative hypothesis is that GBP’s main action mechanism is increasing the expression of extrasynaptic δ subunit-containing GABA A (δGABAA) receptors in neurons, increasing a tonic inhibitory conductance in neurons. The enhanced δGABAA receptor function is believed to contribute to the ataxic and anxiolytic effects but does not explain the antinociceptive properties of GBP [11].

GBP side effects include fatigue, ataxia, headache, weight gain, diarrhea, diplopia, dizziness, leg edema, tremor, and vomiting [12]. In the U.S. Gabapentin Study Group 1993, adverse events were reported by 88% of GBP users. Central nervous system effects that occurred in more than ten percent of patients included nystagmus (19%), somnolence, diplopia, tremor (15%), ataxia (14%), and dizziness (13%). Only 7% of all first occurrences of adverse events were of severe intensity [6]. Movement disorders secondary to GBP were rarely described in the initial clinical trials of this drug. Also, the first reports of abnormal movements besides tremors and ataxia were only observed after the approval of GBP for marketing. In this context, the present literature review aims to evaluate the clinical–epidemiological profile, pathological mechanisms, and management of GBP-associated movement disorder (MD). To the authors’ knowledge, this is the first publication with a systematic approach to assessing the MDs associated with GBP.

## 2. Materials and Methods

### 2.1. Search Strategy

We searched six databases to locate all the existing reports on MDs secondary to GBP published from 1990 until June 2023 in electronic form. Excerpta Medica (Embase), Google Scholar, Latin American and Caribbean Health Sciences Literature (Lilacs), Medline, Scientific Electronic Library Online (Scielo), and Science Direct were searched. Search terms were “akathisia, ataxia, ballism, bradykinesia, chorea, dyskinesia, dystonia, extrapyramidal, hyperkinetic, hypokinetic, movement disorder, myoclonus, myokymia, parkinsonism, restless legs syndrome, restlessness, stuttering, tics, tremor”. These terms were combined with “gabapentin” (Table 1).

### 2.2. Inclusion and Exclusion Criteria

The inclusion criteria covered case reports, case series, original articles, letters to the editor, bulletins, and poster presentations, published from 1990 to June 2023, without language restriction to ensure a thorough review. In the cases where the non-English literature was beyond the authors’ proficiency (English, French, and Spanish) or when the English abstract did not provide enough data, such as articles in Chinese, Japanese, and Korean, the Google Translate service was used [13].

The authors independently screened the titles and abstracts of all articles from the initial search. Disagreements between authors were solved through discussion. Cases where the cause of MD was already known and the motor symptoms were not worsened or unrelated to GBP were excluded. Also, cases not accessible by electronic methods, including after a formal request to the authors, were excluded. Cases with more than one factor contributing to the MD were evaluated based on the probability of the event occurrence based on the Naranjo algorithm.

### 2.3. Data Extraction

**Figure 2:**
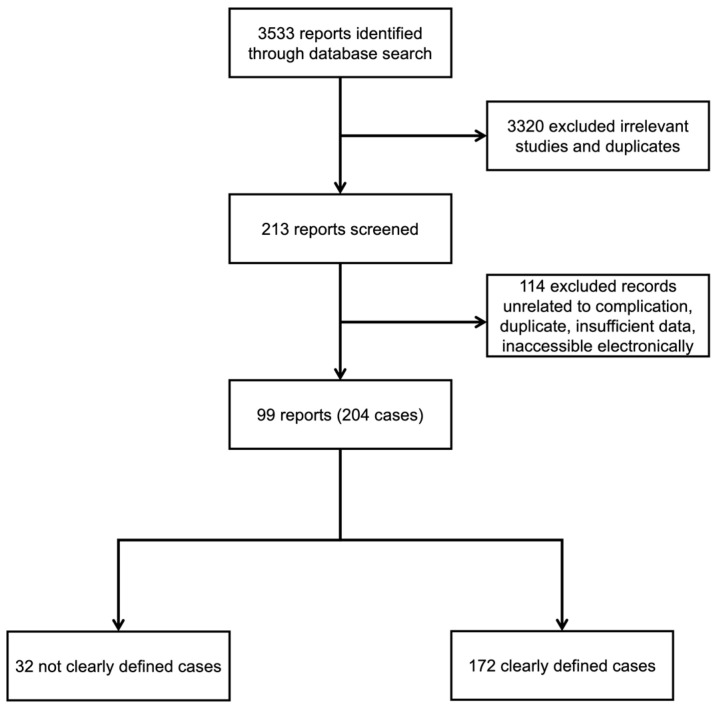
Flowchart of the screening process.

A total of 3533 articles were found; 3320 were inappropriate, and 114 were unrelated to the subject, duplicates, inaccessible electronically, or provided insufficient data (Figure 2). Data abstraction was performed. When provided, we extracted author, department, year of publication, country of occurrence, number of patients affected, GBP indication including off-label uses, time from first GBP dose until MD onset, time from GBP withdrawal to symptoms improvement, patient’s status at follow-up, and significant findings of clinical history and management. The data were extracted by two independent authors and double-checked to ensure matching.

### 2.4. Statistical Analysis

Categorical variables were represented as proportions. Continuous variables were represented as means, standard deviation (SD), median, and range. Statistical analysis was performed using Microsoft Excel Spreadsheet Software version 16.0 (Microsoft Corp, Redmond, WA, USA).

### 2.5. Definitions

The clinical characteristics and definitions of the MDs, such as parkinsonism, tics, dyskinesia, dystonia, stuttering, myoclonus, restless legs syndrome, akathisia, tremors, chorea, ataxia, and ballism, were obtained from Jankovic and colleagues [14]. The clinical diagnosis for the psychiatric conditions was obtained from the *Diagnostic and Statistical Manual of Mental Disorders* (Fifth Edition) [15]. The Naranjo algorithm was used to determine the likelihood of whether an adverse drug reaction was actually due to the drug rather than the result of other factors [16]. Movement disorder onset was defined as the time from the GBP start until the development of the movement disorder. Movement disorder recovery was defined as the time from the first management, which could be the GBP discontinuation, until the full recovery of the abnormal movement [17].

## 3. Results

**Figure 3:**
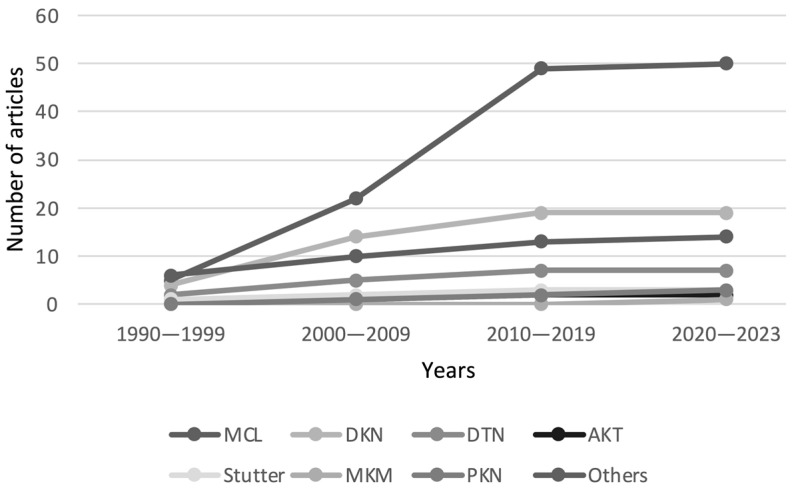
Line graph showing the cumulative number of publications regarding gabapentin-associated movement disorder from 1990 to 2023. AKT, akathisia; DKN, dyskinesia; DTN, dystonia; MCL, myoclonus; MKM, myokymia; PKN, parkinsonism. In the “Others” subgroup are cases not specified about the movement disorder, such as extrapyramidal symptoms.

Between 1990 and 2023 (June), 99 reports of 204 individuals who developed a MD associated with GBP were identified from 21 countries. Ninety-one subjects were from North American countries, 81 European, 30 Asian, 1 South American, and 1 Australian. Figure 3 shows the number of articles published about MDs and GBP over time (Figure 3). The MDs associated with GBP encountered were 135 myoclonus, 22 dyskinesias, 7 dystonia, 3 akathisia, 3 stutterings, 1 myokymia, and 1 parkinsonism. In the subgroup of cases not clearly defined, there were 32 individuals (Table 2 and Table 3).

The summary data about GBP-associated MDs are provided in Table 4. Herein, we describe the general data of all clearly defined cases.

The mean and median ages were 54.54 (SD: 17.79) and 57 years (age range: 10–89), respectively. Subjects were slightly predominantly male, corresponding to 53.57% (60/112) of the individuals. It is worth mentioning that in 92 individuals, the sex was not defined. The most common indication of GBP was neuropathic pain [101]. Some other GBP uses were focal epilepsy, generalized epilepsy, focal-generalized epilepsy [20], essential tremors [97], restless legs syndrome [85], generalized anxiety disorder [82], and paroxysmal kinesigenic dyskinesia [83]. The mean and median doses of GBP when the MD occurred were 1324.66 (SD: 1117.66) and 1033 mg/daily (GBP dose range: 100–9600), respectively).

The mean and median times from GBP-onset to GBP-associated MD were 4.58 (SD: 8.08) and one week (MD onset range: 1 h to 4 years). The mean and median recovery times after MD treatment were 4.17 (SD: 4.87) and 2.5 days (MD recovery range: 1 day to 12 months) in cases who achieved complete remission of the symptoms.

The MD management involved GBP discontinuation. In elevated GBP concentrations, hemodialysis [36] and peritoneal dialysis [39] were performed. Benzodiazepines were attempted to alleviate myoclonus [38]. In the individuals who developed dyskinesia, diphenhydramine was started [72]. Also, some authors described GBP rechallenges and demonstrated MD’s reoccurrence [73]. There was no report of death associated with GBP use. A total of 82.5% of the individuals had a full recovery in the follow-up period.

## 4. Discussion

### 4.1. General

Gabapentin (GBP) is U.S. FDA-approved for the adjunctive treatment of focal seizures and the treatment of postherpetic neuralgia. GBP also exhibits analgesic properties, often used as the first line in managing neuropathic pain. GBP-induced movement disorders are under-recognized adverse drug reactions. They are commonly not discussed with patients, and their sudden occurrence can lead to misdiagnosis of a “seizure-like” condition. Also, first-contact physicians might treat them as seizures or psychiatric comorbidities, leading to unnecessary tests and aggressive management.

Gabapentin is among the ten most commonly prescribed medications in the United States. A total of 49,961,066 prescriptions of GBP were issued in 2020 [115]. It has been available as a generic medication in the United States since 2004. During the 1990s, Parke-Davis, a subsidiary of Pfizer, used many illegal techniques to encourage physicians in the U.S. to prescribe GBP for unapproved uses [116]. Also, there are some concerns regarding gabapentin’s off-label uses due to the lack of strong scientific evidence for its efficacy in multiple conditions and its proven side effects [117]. Moreover, GBP is not scheduled or considered a controlled substance (as per the Controlled Substances Act) at the federal level. But, some states, like Kentucky and Michigan, have classified gabapentin as a Schedule V controlled substance [118].

Based on the results, we could illustrate the average person who developed a MD secondary to GBP as a middle-aged male of North American origin who used a GBP dose from 1200 to 1500 mg daily prescribed for managing his neuropathic pain. This individual presented with multifocal large-amplitude myoclonic jerks, mainly in the distal extremities, with onset after four weeks of the GBP start. The management was medication withdrawal, and a full recovery was observed within a week.

Significant and recurrent features are observed in the description of drug-induced MDs that lead to a poor understanding of this condition. First, most manuscripts do not describe motor symptoms, which can lead to misinterpretations. Recording methods of abnormal movements can decrease the influence of this drawback and provide further observations for future analysis. Second, there is a significant lack of supporting studies, especially for medications with many patients presenting with myoclonus, such as GBP. Therefore, no myoclonic source can be identified in most cases.

Table 5 describes the incidence of some abnormal movements associated with GBP in the literature (Table 5). In a retrospective study, one hundred patients with epilepsy were adjuvanted with GBP for at least three months. Seventy-two patients experienced a greater than 50% seizure reduction. Thirty-five patients reported 43 side effects. Twenty-six side effects took place at 900 mg or less dosages from these. Seven patients taking a mean dosage of 1485 mg/d reported ataxia (carbamazepine was concomitant therapy in six patients). Orofacial dyskinesia happened in one patient, and tremors also occurred in only one patient [12]. In the U.S. Gabapentin Study Group, of 240 patients, 88% experienced adverse events. The mean dosages were, on average, 1200–2400 mg/day. In these patients, central nervous system effects occurred in more than 10%. In this subgroup, 19% had nystagmus, 15% had diplopia, 15% had tremors, and 14% presented ataxia [6].

**Figure 4:**
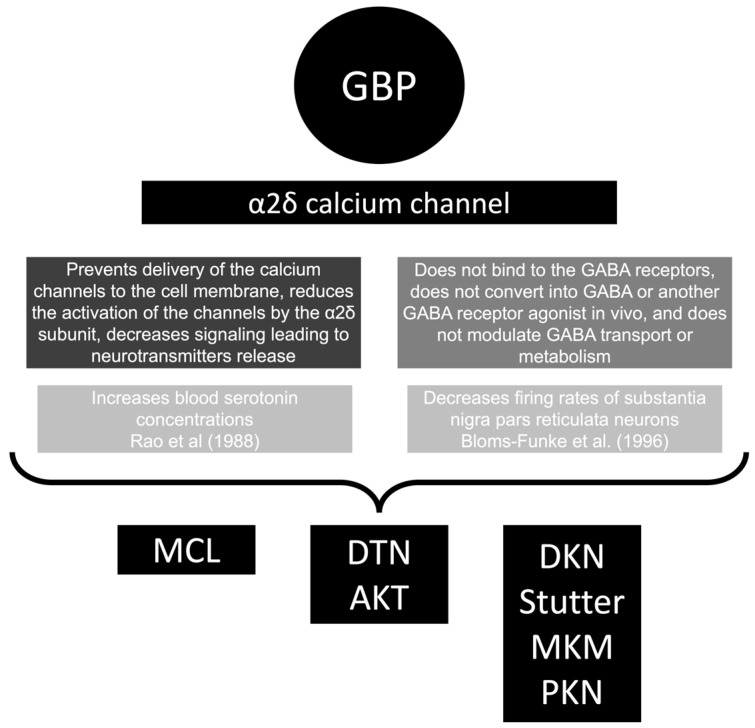
Schematic diagram of the pathophysiological mechanism for gabapentin (GBP)-associated movement disorder. AKT, akathisia; DKN, dyskinesia; DTN, dystonia; GABA, gamma-aminobutyric acid; MCL, myoclonus; MKM, myokymia; PKN, parkinsonism [119,120].

Herein, we would like to discuss some of the MDs in subtopics to allow a better comprehension of the data (Figure 4) [119,120].

### 4.2. Myoclonus

Myoclonus was the most common movement disorder described with GBP, corresponding to 66.17% of the cases. The incidence of myoclonus varied from 1.09 to 21.05%, and the incidence was not affected by sex. The presentation of GBP-induced myoclonus was generalized myoclonus [19], multifocal [28], and focal [64]. A paradoxical aggravation of benign adult familial myoclonic epilepsy was reported in the literature [33]. Interestingly, some individuals presented with frequent unexplained falls, which can sometimes be misinterpreted in mental health settings, with hypotension or sedation caused by other medications [27]. Noteworthy, is that Hui et al. reported a case of jaw myoclonus leading to dysphagia with an inability to drink liquids [64]. Three patients from the Desai et al. case series that were treated with gabapentin had myoclonic jerks on the side contralateral to their epileptic focus. This finding may suggest that brain areas with antecedent dysfunction are more vulnerable to the toxic effects of calcium channel blockers [63].

Myoclonic symptoms can lead to significant distress or impaired function. One patient developed a myoclonus interfering with ambulation [27], and another had his speech impacted by the myoclonus of the lower facial muscles [64]. However, there were cases where myoclonic symptoms were tolerable and did not affect daily living activities, going clinically unnoticed without specific questioning [24].

Some, but not all, patients who experienced myoclonus presented with altered mental status. A toxic encephalopathy was observed in individuals who developed negative myoclonus secondary to gabapentin [26]. Chau et al. reported a patient with Creutzfeldt–Jakob disease-like syndrome induced by gabapentin toxicity, in which electrodiagnostic studies showed periodic sharp wave complexes [40]. A similar clinical manifestation with electrodiagnostic studies was already observed with amitriptyline [121] and fluoroquinolones [17].

The neurophysiological source was cortical and subcortical. Interestingly, Scheyer et al. reported a reflexive response of myoclonus only with high doses of GBP [20]. Therefore, the source of myoclonus can be directly associated with the concentration of GBP. However, most articles did not report electrodiagnostic studies or a detailed clinical description of myoclonus for a clear diagnostic assumption of this hypothesis. Pierce et al. reported a 46-year-old female presenting with a triad of hearing loss, myoclonus, and confusion. GBP concentration was elevated, and hemodialysis was performed with complete recovery of the neurological symptoms [36]. This interesting case shows a possible association between cortical and subcortical myoclonic sources [122]. One probable cause of hearing loss in this individual could be the tensor tympani myoclonus [123].

Reeves et al. reported an association between myoclonus frequency and GBP dose [19]. Hence, we can hypothesize that the GBP-induced myoclonus is more likely to be a linear dose-dependent adverse effect rather than a threshold effect. It is worth mentioning that GBP exhibits nonlinear (zero-order) kinetics due to the saturable absorption [124]. Therefore, levels of GBP do not increase in proportion with increasing doses. In this context, Healy et al. reported a patient having myoclonus with GBP and pregabalin on different occasions [37].

One important fact to mention was the association between possible intoxication doses of GBP and kidney failure. Zhang et al. considered renal dysfunction a significant risk factor for developing myoclonus associated with GBP [29]. The case reported by Holtkamp et al. can support this hypothesis because myoclonus only occurred when the individual renal function became worse [32]. However, GBP-induced myoclonus was already observed in previously healthy individuals [34]. Noteworthy, GBP does not undergo hepatic metabolism and is primarily excreted unchanged in the urine. Therefore, renal excretion is the primary pathway for the systemic elimination of GBP, and subjects with poor renal clearance require dose adjustment to prevent elevated plasma concentrations and adverse side effects [125]. Refractory epilepsy [21], diffuse brain damage [26], higher doses of GBP [36], and renal function [43] were the most commonly reported risk factors for developing GBP-induced myoclonus. Individual differences in the occurrence of myoclonic jerks with GBP may be based on genetics or co-morbidities.

The mechanism of GBP-induced myoclonus is probably related to the serotoninergic hypothesis similar to that proposed for buspirone-associated movement disorders [126]. Klawans et al. induced myoclonus in young guinea pigs by administering the serotonin precursor, 5-hydroxytryptophan [127]. Serotonin blood levels are increased in healthy people exposed to GBP [119]. But, there is no evidence of GBP directly acting on serotonin receptors [128].

Huppertz et al. reported that, in GBP-induced myoclonus, the therapeutical dose of GBP should be maintained because the myoclonus will eventually improve with time [24]. But, some authors attempted to reintroduce GBP, and myoclonus reappeared [44]. Ahmad et al. stated that a slow de-escalation should be conducted to avoid developing chronic side effects [58]. Myoclonus secondary to GBP had the worst prognosis among the GBP-induced movement disorders, with only 79.24% of the individuals fully recovering.

### 4.3. Dyskinesia

The presentation of GBP-induced dyskinesia was ballism [84], chorea [79], hemichorea [78], choreoathetosis [71], and orofacial dyskinesia [75]. Shin et al. reported a 44-year-old male with chorea who had worsened choreiform movements after administering GBP for his restless legs syndrome [79]. Bonakis et al. described a similar case in which the individual was diagnosed with paroxysmal kinesigenic dyskinesia worsened by GBP [83].

The occurrence of dyskinesia associated with GBP widely varied in the literature. Choreoathetosis was observed in 7.1% of the GBP users when this side effect was a secondary outcome [74]. Some risk factors proposed for GBP-induced dyskinesia were cognitive impairment [73] and a history of previous movement disorder [77].

Aksoy et al. reported an interesting case of a 70-year-old male presenting with generalized choreiform movements with pramipexole, pregabalin, and gabapentin on different occasions [85]. This case can support a similar mechanistic hypothesis for these drugs leading to chorea, which can be associated with a dopaminergic pathway. Another possible mechanism is related to the GABAergic effect. In rat models, GBP decreased the activity of GABA neurons in the substantia nigra [120]. It is important to note that not all patients who take GBP experience chorea. This suggests that certain patients may have unique neurophysiological factors that make them more susceptible to this side effect.

The most common management was the discontinuation of the offending drug. Some authors reported administering other drugs to improve dyskinetic symptoms, such as diphenhydramine [72]. The full recovery rate of dyskinesia associated with GBP was 88.23%, above the mean data for GBP-induced movement disorders.

### 4.4. Dystonia

Dystonia was the third movement disorder most commonly reported with GBP. Interestingly, the population affected by GBP-induced dystonia had a lower mean age when compared to the other GBP-induced movement disorders. It is worth mentioning that this is a common finding among drug-induced movement disorders [129,130]. Most individuals presented with segmental dystonia [95], but status dystonicus [19], generalized dystonia [96], and axial dystonia [98] were also observed. Alford et al. described an individual who developed myotonia associated with dystonia [98].

Reeves et al. reported a case of a 23-year-old female with a history of epilepsy who was on carbamazepine and was prescribed GBP for panic attacks. The patient reported muscle twitches after using 1200 mg/day of GBP for four days. Her movements were of small amplitude and possibly dystonic or myoclonic. Reeves et al. described another patient with drug-resistant epilepsy who was on GBP 600 mg three times a day for one month and suddenly presented an oculogyric crisis, retrocollis, opisthotonic posturing, and repetitive jaw clenching. Electroencephalogram monitoring was normal. He was treated with 2 mg IV lorazepam, and his abnormal movements ceased after being present for approximately 15 h. After GBP discontinuation, the movements did not recur [19]. Both reports by Reeves et al. are important because the individuals had a delayed diagnosis due to being misdiagnosed as having a psychiatric disorder [131].

Apparently, the GBP dose is related to the occurrence of dystonia [95]. Also, some authors reported a synergistic effect with some medications, such as propranolol [96] and anesthetic agents [98]. Rohman et al. described a 26-year-old male who used a GBP single dose of 1600 mg for recreational abuse and developed cervical dystonia [100]. There has been a concerning increase in the abuse of GBP, and previously unknown adverse effects of this medication are being discovered. Recently, there have been reports of lethal overdoses caused by this once-believed harmless drug [132]. Therefore, the study by Rohman et al. provides a new possible clinical manifestation to be observed when there is suspicion of recreational abuse of GBP.

A proposed mechanism for GBP-induced dystonia is the abnormal concentrations of different monoamines. It was already observed that catecholamine depletion by alpha-methyl-para-tyrosine can cause acute dystonia in healthy volunteers [133]. Bernal et al. proposed that the dystonia observed with GBP was probably related to an increased GABAergic effect leading to decreased paroxysmal discharges, causing an increased dopaminergic effect [95]. Another possible explanation can be related to the increased serotonin levels observed with GBP [119]. An association between dystonia and the serotonergic system was already reported due to basal ganglia serotonin and serotonin–dopamine interactions [134].

The management was GBP discontinuation and lorazepam [19] or procyclidine intravenously [100] to shorten the recovery time. Palomeras et al. reported improved dystonic symptoms after reducing the adjunctive drug without changing the GBP dose [96]. A total of 83.33% of the patients with PGB-induced dystonia had a full recovery. One patient did not recover, and the persistence of his abnormal movements in the upper right limb throughout the follow-up was observed [95].

### 4.5. Akathisia

Childers et al. reported two cases of akathisia secondary to GBP therapy. Both patients used GBP 900 mg/day and fully recovered within two days of GBP discontinuation. The authors stated that brain-injured individuals are more sensitive to GBP therapy; consequently, this subgroup of GBP users more commonly develops side effects with even lower doses of GBP [90]. See et al. described a 76-year-old female with neuropathic pain managed with GBP 3600 mg/day. The patient developed akathisia due to GBP withdrawal, which is a unique report because this is the only report of a movement disorder, besides tremors and ataxia, associated with GBP withdrawal syndrome [91]. Possible explanations for GBP-induced akathisia could be related to the serotoninergic and dopaminergic hypothesis. Noteworthy, is that GBP does not affect 5HT2A commonly reported receptor associated with drug-induced akathisia [135]. Interestingly, GBP was already reported to improve akathisia symptoms caused by antipsychotics [136,137].

### 4.6. Stuttering

The occurrence of stuttering associated with GBP has been rarely reported. Interestingly, all the patients with stuttering following GBP therapy fully recovered after drug discontinuation. Nissani et al. probably reported the first case of stuttering secondary to GBP. According to the authors, the patient was a middle-aged female with epilepsy, managed with phenytoin. She was then admitted for an “intractable seizure” when GBP was started, after which she developed stuttering. Four days after GBP discontinuation, the stuttering resolved [92]. In another case report, a 66-year-old man with postherpetic neuralgia was managed with GBP. After some days, the patient complained of stuttering, and GBP therapy was discontinued, with complete recovery of the symptoms [93]. Zeldin et al. reported a 62-year-old male with chronic back pain for whom methylprednisolone and GBP 300 mg thrice daily were started. After two days on this drug regimen, the patient presented stuttering, which started twenty minutes after taking GBP. Neuroimaging was unremarkable. GBP was gradually tapered, and the stuttering improved [94].

Stuttering is believed to occur due to hyperactivity in the brain’s right hemisphere, abnormal coordination between speech planning and execution centers, and abnormal dopamine concentrations [138]. Animal models showed that GBP causes abnormal dopamine concentrations in the striatum [139]. In this way, the abnormal levels of this neurotransmitter may lead to dysfunction between the motor cortex and Broca’s area. Pregabalin, another GABA analog, was already observed with stuttering [140]. Therefore, this side effect may be a class effect of GABA analogs. Interestingly, GBP was also effective in managing neurogenic stuttering [141]. Therefore, GBP may provoke or reduce stuttering, which the multiple interacting neurotransmitter systems can explain.

### 4.7. Myokymia and Parkinsonism 

Only one study reported myokymia associated with GBP use, but the drug was probably at toxic levels. The patient was a 69-year-old man taking GBP in a prescribed dosage of 9600 mg/day. He had a previous history of peripheral neuropathy, traumatic brain injury, amnesia, and post-traumatic stress disorder. He presented with muscle spasms and falls. He had focal and segmental myokymia observed in his lower extremities, which were worse in the calves than in the thighs. The GBP level was 25.8 μg/dL (therapeutic range, 2–20 mcg/mL). After reducing the daily dosage of GBP, the patient presented improvement in his abnormal movements [101]. Elevated serotonin levels may partially explain GBP-induced myokymia. Noteworthy, is that flunarizine, a calcium channel inhibitor, shares a similar mechanism of action with GBP. And, this drug was already reported to cause myokymia [142]. Although GBP-induced myokymia is scarce, there are many reports about myokymia managed with GBP. Superior oblique myokymia was already observed to improve with GBP therapy [143]. GBP is believed to alleviate myokymia by causing a membrane-stabilizing effect that avoids peripheral nerve excitability [144].

Gabapentinoids affect several neurotransmitters, including dopamine and serotonin. It is believed that their chronic effect may increase the risk of parkinsonism. Pacheco-Paez et al. assessed 5,653,547 reports, of which 4881 individuals with Parkinson’s disease had a chronic use of GBP. The authors reported an increased odds ratio (OR = 2.16, 95% CI 2.10–2.23) of Parkinson’s disease in GBP users [69]. Ri et al. performed a case-crossover study in the Japanese population to assess the risk of parkinsonism with gabapentinoids. They observed that exposure to gabapentinoids (adjusted OR, 2.12; 95% CI, 1.73–2.61) was associated with an increased risk of developing parkinsonism [70].

### 4.8. Gabapentin and Pregabalin-Associated Movement Disorders

Pharmacologically, GBP and pregabalin bind to the alpha-2-delta protein, a subunit of the voltage-gated calcium channels and a receptor involved in regulating neuronal excitability. The inhibition of alpha-2-delta receptors decreases calcium influx at the nerve terminal, reducing the release of excitatory neurotransmitters (glutamate, noradrenaline), thereby reducing pain signaling. Here, we would like to compare the epidemiological profile of the individuals affected by GBP and pregabalin-associated movement disorders (Table 6) [140].

There was a similar prevalence among the most commonly reported movement disorders associated with GBP and pregabalin. After ataxia and tremors, commonly reported adverse events without clear clinical specification in clinical trials, myoclonus for both drugs was the most common abnormal movement reported. One interesting fact to mention regarding myoclonus and these gabapentinoids drugs is the fact that gabapentin has risk factors that are usually observed in the population affected, such as refractory epilepsy, diffuse brain damage, and renal function. But, pregabalin-induced myoclonus does not have significant risk factors.

Interestingly, GBP is more commonly reported with dystonia and dyskinesia. On the other hand, pregabalin was more frequently described with parkinsonism. Recent studies assess the risk of gabapentinoids as a class in developing Parkinson’s disease. Therefore, based on this present data, future studies should assess the effect of GBP and pregabalin with sensitive analysis because pregabalin may be a major risk factor than GBP for the development of Parkinson’s disease.

The individuals that developed a movement disorder secondary to GBP had the worst prognosis than those who had one secondary to pregabalin. Noteworthy, is that the description of the follow-up of pregabalin-induced movement disorder was only provided by 18 articles. Therefore, further assumptions regarding the follow-up of pregabalin-associated movement disorder cannot be provided.

## 5. Limitations

There are some limitations in the present study. First, we included different types of studies to review the literature thoroughly. But, this can affect the incidence and prevalence among the different movement disorders described. Also, ataxia and tremors were not included in the analysis because these movement disorders affected more than ten percent of the GBP users [6]. Third, most of the reports did not describe electrodiagnostic or neuroimaging studies. Fourth, the clinical description of the abnormal movements was vague, and movement disorders specialists described fewer reports, which can affect the diagnosis of the abnormal movement. GBP-induced myoclonus was described in more detail than other movement disorders, probably not only by the incidence of this abnormal movement with GBP but also due to the trending of publications. This could have affected the overall description of other GBP-induced movement disorders.

Myoclonus is the only movement disorder secondary to GBP with high evidence (GRADE A) when we use the Grading of Recommendations, Assessment, Development, and Evaluations (GRADE) system. GBP-induced dyskinesia probably has a low level of evidence (GRADE C). However, all the other movements (dystonia, akathisia, stutter, myokymia, and parkinsonism) associated with GBP also have low evidence (GRADE D).

## 6. Conclusions

In summary, the movement disorders encountered with GBP were, in order of frequency, myoclonus, dyskinesias, dystonia, akathisia, stuttering, myokymia, and parkinsonism. The generic availability and wide off-label use probably contributed to the occurrence of these uncommon reports. The abnormal movements are likely associated with the serotoninergic hypothesis and the dysfunction in the substantia nigra caused by GBP. The most common management was GBP withdrawal, but some authors proposed maintaining GBP therapy because the motor symptoms would eventually improve. There are interesting similarities and differences between GBP and pregabalin that were highlighted.

## Figures and Tables

**Table 1:** FreeText and MeSH search terms in the U.S. National Library of Medicine.

Category	Search Terms	Results
Akathisia	(“gabapentin”[MeSH Terms] OR “gabapentin”[All Fields] OR “gabapentine”[All Fields] OR “gabapentin s”[All Fields]) AND (“akathisias”[All Fields] OR “psychomotor agitation”[MeSH Terms] OR (“psychomotor”[All Fields] AND “agitation”[All Fields]) OR “psychomotor agitation”[All Fields] OR “akathisia”[All Fields])	30
Ataxia	(“gabapentin”[MeSH Terms] OR “gabapentin”[All Fields] OR “gabapentine”[All Fields] OR “gabapentin s”[All Fields]) AND (“ataxia”[MeSH Terms] OR “ataxia”[All Fields] OR “ataxias”[All Fields])	132
Ballism	(“gabapentin”[MeSH Terms] OR “gabapentin”[All Fields] OR “gabapentine”[All Fields] OR “gabapentin s”[All Fields]) AND (“dyskinesias”[MeSH Terms] OR “dyskinesias”[All Fields] OR “ballism”[All Fields])	193
Bradykinesia	(“gabapentin”[MeSH Terms] OR “gabapentin”[All Fields] OR “gabapentine”[All Fields] OR “gabapentin s”[All Fields]) AND (“hypokinesia”[MeSH Terms] OR “hypokinesia”[All Fields] OR “bradykinesia”[All Fields])	4
Chorea	(“gabapentin”[MeSH Terms] OR “gabapentin”[All Fields] OR “gabapentine”[All Fields] OR “gabapentin s”[All Fields]) AND (“chorea”[MeSH Terms] OR “chorea”[All Fields] OR “choreas”[All Fields])	26
Dyskinesia	(“gabapentin”[MeSH Terms] OR “gabapentin”[All Fields] OR “gabapentine”[All Fields] OR “gabapentin s”[All Fields]) AND (“dyskinesiae”[All Fields] OR “dyskinesias”[MeSH Terms] OR “dyskinesias”[All Fields] OR “dyskinesia”[All Fields])	208
Dystonia	(“gabapentin”[MeSH Terms] OR “gabapentin”[All Fields] OR “gabapentine”[All Fields] OR “gabapentin s”[All Fields]) AND (“dystonia”[MeSH Terms] OR “dystonia”[All Fields] OR “dystonias”[All Fields] OR “dystonic disorders”[MeSH Terms] OR (“dystonic”[All Fields] AND “disorders”[All Fields]) OR “dystonic disorders”[All Fields])	49
Extrapyramidal	(“gabapentin”[MeSH Terms] OR “gabapentin”[All Fields] OR “gabapentine”[All Fields] OR “gabapentin s”[All Fields]) AND “extrapyramidal”[All Fields]	6
Hyperkinetic	(“gabapentin”[MeSH Terms] OR “gabapentin”[All Fields] OR “gabapentine”[All Fields] OR “gabapentin s”[All Fields]) AND (“hyperkinetic”[All Fields] OR “hyperkinetics”[All Fields])	5
Hypokinetic	(“gabapentin”[MeSH Terms] OR “gabapentin”[All Fields] OR “gabapentine”[All Fields] OR “gabapentin s”[All Fields]) AND (“hypokinesia”[MeSH Terms] OR “hypokinesia”[All Fields] OR “hypokinetic”[All Fields])	1
Movement disorder	(“gabapentin”[MeSH Terms] OR “gabapentin”[All Fields] OR “gabapentine”[All Fields] OR “gabapentin s”[All Fields]) AND (“movement disorders”[MeSH Terms] OR (“movement”[All Fields] AND “disorders”[All Fields]) OR “movement disorders”[All Fields] OR (“movement”[All Fields] AND “disorder”[All Fields]) OR “movement disorder”[All Fields])	224
Myoclonus	(“gabapentin”[MeSH Terms] OR “gabapentin”[All Fields] OR “gabapentine”[All Fields] OR “gabapentin s”[All Fields]) AND (“myoclonus”[MeSH Terms] OR “myoclonus”[All Fields])	80
Myokymia	(“gabapentin”[MeSH Terms] OR “gabapentin”[All Fields] OR “gabapentine”[All Fields] OR “gabapentin s”[All Fields]) AND (“myokymia”[MeSH Terms] OR “myokymia”[All Fields] OR “myokymias”[All Fields])	12
Parkinsonism	(“gabapentin”[MeSH Terms] OR “gabapentin”[All Fields] OR “gabapentine”[All Fields] OR “gabapentin s”[All Fields]) AND (“parkinson disease”[MeSH Terms] OR (“parkinson”[All Fields] AND “disease”[All Fields]) OR “parkinson disease”[All Fields] OR “parkinsons”[All Fields] OR “parkinson”[All Fields] OR “parkinson s”[All Fields] OR “parkinsonian disorders”[MeSH Terms] OR (“parkinsonian”[All Fields] AND “disorders”[All Fields]) OR “parkinsonian disorders”[All Fields] OR “parkinsonism”[All Fields] OR “parkinsonisms”[All Fields] OR “parkinsons s”[All Fields])	82
Restless legs syndrome	(“gabapentin”[MeSH Terms] OR “gabapentin”[All Fields] OR “gabapentine”[All Fields] OR “gabapentin s”[All Fields]) AND (“restless legs syndrome”[MeSH Terms] OR (“restless”[All Fields] AND “legs”[All Fields] AND “syndrome”[All Fields]) OR “restless legs syndrome”[All Fields])	203
Restlessness	(“gabapentin”[MeSH Terms] OR “gabapentin”[All Fields] OR “gabapentine”[All Fields] OR “gabapentin s”[All Fields]) AND (“psychomotor agitation”[MeSH Terms] OR (“psychomotor”[All Fields] AND “agitation”[All Fields]) OR “psychomotor agitation”[All Fields] OR “restlessness”[All Fields] OR “restless”[All Fields])	240
Stuttering	(“gabapentin”[MeSH Terms] OR “gabapentin”[All Fields] OR “gabapentine”[All Fields] OR “gabapentin s”[All Fields]) AND (“stammerers”[All Fields] OR “stammers”[All Fields] OR “stutterer”[All Fields] OR “stutterer s”[All Fields] OR “stutterers”[All Fields] OR “stuttering”[MeSH Terms] OR “stuttering”[All Fields] OR “stammer”[All Fields] OR “stammering”[All Fields] OR “stutter”[All Fields] OR “stuttered”[All Fields] OR “stutters”[All Fields] OR “stutterings”[All Fields])	5
Tics	(“gabapentin”[MeSH Terms] OR “gabapentin”[All Fields] OR “gabapentine”[All Fields] OR “gabapentin s”[All Fields]) AND (“tics”[MeSH Terms] OR “tics”[All Fields])	0
Tremor	(“gabapentin”[MeSH Terms] OR “gabapentin”[All Fields] OR “gabapentine”[All Fields] OR “gabapentin s”[All Fields]) AND (“tremor”[MeSH Terms] OR “tremor”[All Fields] OR “tremors”[All Fields] OR “tremoring”[All Fields] OR “tremorous”[All Fields])	133

**Table 2:** Literature review of gabapentin-associated movement disorders.

Reference	N	Age (Years); Sex	GBP-Indication	GBP-Dose (mg/day)	MD Onset ^a^	MD Recovery ^b^	Follow-Up	CT, MRI; EEG, EMG
Myoclonus								
Asconape et al. (1995) [18]	1	NA	NA	NA	NA	NA	NA	NA
Reeves et al. (1996) [19]	1	23; F	Complex partial seizures	900	2 weeks and 4 weeks	4 days	CR	NA
Scheyer et al. (1996) [20]	8	NA	5 focal epilepsy, 2 generalized epilepsy, 1 focal-generalized epilepsy	2170 (mean)	NA	NA	CR	NA; EEG available in 4 patients only—normal
Wilson et al. (1998) * [21]	2	NA	Refractory partial epilepsy	>3600 mg/day	NA	NA	NA	NA
Asconape et al. (2000) * [22]	13	29.2 (mean); 9F, 4M	Refractory epilepsy	2000 (mean)	8 weeks (mean)	2–12 months (range)	2 CR	NA; EEG performed in 3 patients showed no correlation with MCL
Jacob et al. (2000) [23]	1	60; F	Postherpetic neuralgia	First 2 days: 300 mg; other days: 900 mg a day	4 days	3 days	CR	Cranial CT scan: normal; NA
Huppertz et al. (2001) [24]	4	33.25 (mean); 2F, 2M	Simple and complex partial seizures	312.5 (mean)	NA	NA	CR	NA; EEG and EMG: normal
Scullin et al. (2003) [25]	2	50.5 (mean); 1F, 1M	Neuropathic pain	1500 (mean)	NA	NA	CR	NA
Sechi et al. (2004) [26]	2	76.5 (mean); 2F	Simple partial seizure, DM polineuropathy	900 to 3600	Weeks–2 months	11 days (mean)	CR	Brain MRI: unspecific; EEG: normal; EMG: asterixis
Babiy et al. (2005) [27]	1	74; F	Neuropathic pain	600	Several months	NA	CR	NA; EMG: asterixis
Bookwalter et al. (2005) [28]	1	75; M	Postherpetic neuralgia	3200	1 day	1 day	CR	NA
Zhang et al. (2005) [29]	3	52.6 (mean); 1F, 2M	Muscle spasm, chronic paresthesia, arm pain	1033 (mean)	4 months	4–15 days	CR	NA; EEG: normal
Brefel-Courbon et al. (2006) * [30]	26	NA	NA	NA	NA	NA	NA	NA
Han et al. (2006) [31]	1	57; F	Legs paresthesia	900	2 days	3 days	CR	NA; EEG: abnormal
Holtkamp et al. (2006) [32]	1	66; M	Painful sensorimotor neuropathy	900	2 years	1 day	CR	NA
Striano et al. (2007) [33]	1	57; M	ET	900	3 days	1 day	CR	Brain MRI: normal; EEG: diffuse theta slowing and multifocal spikes
Cho et al. (2008) [34]	1	69; F	Neuropathic pain	900	1 week	2 days	CR	NA; EEG: normal
Ege et al. (2008) [35]	1	87; M	Neuropathic pain	600	single-dose	20 h	CR	Brain MRI: periventricular ischemia and cortical atrophy; EEG: normal
Pierce et al. (2008) [36]	1	46; F	DM peripheral neuropathy	900	4 years	2 days	CR	NA
Healy et al. (2009) [37]	1	47; M	Neuropathic pain	900	NA	NA	CR	NA
Koide et al. (2009) [38]	3	NA	NA	600–1800	2 weeks (mean)	NA	CR	NA
Onuigbo et al. (2009) [39]	1	NA	NA	NA	NA	3 days	CR	NA
Chau et al. (2010) [40]	1	78; M	Trigeminal neuralgia	100	3 months	2 days	CR	Cranial CT scan: normal; EEG showed diffuse background slowing with larger amplitude delta discharges, which at times appeared triphasic and were interpreted as consistent with periodic sharp wave complexes
Honma et al. (2010) [41]	2	NA; 1F, 1M	Neuropathic pain	200–400	1 day–4 months	1–2 days	CR	NA
Zand et al. (2010) [42]	8	NA	NA	NA	NA	NA	NA	NA
Choe et al. (2011) [43]	1	69; M	NA	1200	2 days	1 week	CR	NA
Kampf et al. (2011) [44]	1	40; F	Chronic pain syndrome	3200	Months	NA	CR	Brain MRI: EEG: slowed background activity with generalized spike-wave-complexes up to 7 s with a density of 0–4/min normal
Prieto-Pérez et al. (2011) [45]	1	77; M	Restless legs syndrome	NA	2 weeks	3 days	CR	Brain MRI: chronic stroke; NA
Guddati et al. (2012) [46]	1	57; M	Neuropathic pain	900	NA	3 days	CR	NA
Torregrosa-Juan et al. (2012) [47]	1	49; NA	Lumbosacral pain	1800	2 days	NA	NA	Brain MRI: normal; NA
Atalay et al. (2013) * [48]	1	NA	NA	NA	NA	NA	NA	NA
Kagnoff et al. (2013) [49]	1	85; M	NA	1200	NA	NA	NA	NA
Wahba et al. (2013) [50]	1	68; M	Neuropathic pain	300	2 days	3 days	CR	NA; EMG: irregular MCL on isometric muscle contraction manifesting as brief interruption of muscle tone for 50 to 200 msec
Wiegand et al. (2013) [51]	4	31.5 (mean); 2F, 2M	Recreational abuse	NA	NA	NA	NA	NA
Kaufman et al. (2013) [52]	2	66.5 (mean); 1F, 1M	Neuropathic pain	600–900	3 days–months	days	CR	NA
Shea et al. (2014) [53]	1	79; F	Postherpetic neuralgia	300	10 h	5 days	CR	NA
Clark et al. (2015) [54]	1	80; F	Postherpetic neuralgia	900	4 days	2 days	CR	NA
Guner et al. (2015) [55]	1	65; M	Neuropathic pain	1200	1 week	2 days	CR	NA
Ozmenoglu et al. (2015) [56]	1	68; M	Neuropathic pain	1800	2 weeks	2 days	CR	NA
Schnitzer et al. (2016) [57]	1	69; F	Neuropathic pain	3200	Days	Days	CR	NA
Ahmad et al. (2017) [58]	3	68 (mean); 3M	NA	900	NA	NA	CR	NA
Khan et al. (2017) [59]	1	55; F	Lumbosacral pain	1500	NA	1 day	CR	Cranial CT scan: normal; EEG: generalized mild–moderate slowing and generalized discharges with triphasic morphology
Kim et al. (2017) [60]	12	62.58 (mean); 4F, 8M	NA	400 (mean)	3.33 (mean)	NA	CR	NA
Zheng et al. (2017) [61]	1	74; F	Neuropathic pain	900	NA	NA	CR	NA
Perez et al. (2018) [62]	1	47; F	Neuropathic pain	2400	NA	3 days	CR	Cranial CT scan: normal; EEG: generalized, slow waveforms consistent with diffuse encephalopathy
Desai et al. (2019) [63]	7	53 (mean); 4F, 3M	Neuropathic pain	NA	NA	NA	CR	NA; Pt 1: Background slow wave activity. MCL captured on EEG. Pt 4: Periodic discharges of triphasic morphology with background slowing. MCL captured on EEG
Hui et al. (2019) [64]	1	89; F	Postherpetic neuralgia	300	2 months	2 days	CR	Cranial CT scan: normal; EEG: abnormal
Medsafe et al. (2019) * [65]	1	35; M	NA	NA	NA	NA	CR	NA
Yeddi et al. (2019) [66]	1	62; F	Painful leg muscle spasms	200	2 days	3 days	CR	Cranial CT scan: normal; EEG: normal
Latief et al. (2021) [67]	1	64; M	Neuropathic pain	300	1 week	NA	NA	NA
Parkinsonism								
Zadikoff et al. (2007) * [68]	1	61; F	Seizure	NA	NA	NA	NA	NA
Pacheco-Paez et al. (2020) * [69]	NA	NA	NA	NA	NA	NA	NA	NA
Ri et al. (2023) * [70]	NA	NA	NA	NA	NA	NA	NA	NA
Dyskinesia								
Buetefisch et al. (1996) [71]	1	NA	NA	NA	NA	NA	NA	NA
Millichap et al. (1996) [72]	1	37; M	Intractable seizure	NA	5 days	2 days	NA	NA
Chudnow et al. (1997) [73]	2	NA	Intractable seizure	1200–1800	NA	NA	CR	NA
Millichap et al. (1997) [74]	2	41.5 (mean); NA	Intractable seizure	1200–1800	14 days	NA	No	
Norton et al. (2001) [75]	2	50.5 (mean); 2M	Anxiety disorder	1050 (mean)	3 days	2 days	CR	Brain MRI: normal
Lai et al. (2007) [76]	1	NA	Neuropathic pain	1200	NA	5 days	CR	NA
Raju et al. (2007) [77]	1	75; M	Neuropathic pain	600	2 weeks	3 days	CR	NA
Lai et al. (2008) [78]	1	41; M	Neuropathic pain	1200	4 days	1 week	CR	NA
Shin et al. (2008) [79]	1	44; M	Peripheral neuroapthy, and RLS	900	NA	NA	NA	NA
Twardowschy et al. (2008) [80]	1	68; F	Neuropathic pain	NA	30 days	1 week	CR	Brain MRI: unspecific
Zesiewicz et al. (2008) [81]	1	46; F	Complex regional pain syndrome type 1	2100	4 weeks	10 days	CR	Brain MRI: normal
Attupurath et al. (2009) [82]	1	75; M	Anxiety disorder	900	>1 month	2 days	CR	Brain MRI: normal; EEG: normal
Bonakis et al. (2009) [83]	1	42; M	Paroxysmal kinesigenic DKN	900	2 days	NA	NA	NA
Erol et al. (2009) [84]	1	83; F	Neuropathic pain	NA	5 days	4 weeks	CR	Cranial CT scan: normal
Aksoy et al. (2013) [85]	1	70; M	Restless legs syndrome	900	NA	NA	NA	Brain MRI: normal
Souzdalnitski et al. (2014) [86]	1	70; F	Lumbosacral pain	900	3 months	Months	CR	Brain MRI: unspecific
VanHook et al. (2017) [87]	1	75; M	Lumbosacral pain	1200	one year	2 days	CR	Brain MRI: normal
Rahil et al. (2018) [88]	1	39; F	Upper limb pain	300	2 days	1 week	CR	Brain MRI: normal
Hampton et al. (2019) [89]	1	82; M	NA	1200	NA	1 day	CR	Cranial CT scan: normal; EEG: normal
Akathisia								
Childers et al. (1997) [90]	2 (CR)	NA	Neuropathic pain	900	1 week	2 days	NA	NA
See et al. (2011) [91]	1	76; F	Neuropathic pain	3600	NA	NA	NA	NA
Stuttering								
Nissani et al. (1997) [92]	1	58; F	Intractable seizure	NA	NA	4 days	CR	NA
Choi et al. (2004) [93]	1	66; M	Postherpetic neuralgia	NA	NA	NA	CR	NA
Zeldin et al. (2019) [94]	1	62; M	Lumbosacral pain	900	2 days	Weeks	CR	Cranial CT scan: normal
Dystonia								
Reeves et al. (1996) [19]	1	24; M	Complex partial seizures	1800	2 months	<1 h	CR	NA; EEG: normal
Bernal et al. (1999) [95]	1	10; M	Behavioral problems	900	16 months	NA	No	NA; EEG normal
Palomeras et al. (2000) [96]	1	68; M	ET	GBP 900 mg/day + propranolol 80 mg/day	2 days when adding propranolol to GBP therapy	20 days	CR	NA
Pina et al. (2005) [97]	1	72; F	ET	2100	NA	Weeks	CR	Cranial CT scan: normal; EEG normal
Allford et al. (2007) [98]	1	55; F	Neuropathic pain	1800	NA	2 days	CR	Brain MRI; EEG normal:
Lal et al. (2011) [99]	1	NA	NA	NA	NA	NA	NA	NA
Rohman et al. (2014) [100]	1	26; M	NA	1600	Single-dose	1 day	CR	NA
Myokymia								
Brown et al. (2021) [101]	1	69; M	Neuropathic pain	9600	NA	3 days	CR	Brain MRI; EEG: normal
Others not clearly defined								
The US Gabapentin Study Group (1994) * [6]	15% developed tremor	NA	NA	NA	NA	NA	NA	NA
Bergey et al. (1997) * [102]	4	NA	NA	NA	NA	NA	NA	NA
Steinhoff et al. (1997) [103]	2	26 (mean); 1F, 1M	Drug-resistant epilepsy	300 (mean)	6 days (mean)	1 day (mean)	CR	Case 1—NA, case 2—MRI revealed cerebellar atrophy mainly of the vermis; Case 1—NA; case 2—frontal lobe origin
Mayer et al. (1999) * [104]	NA	37.6 (mean); NA	Seizure	≤2400	43 days	NA	NA	NA
McLean et al. (1999) * [105]	NA	NA	Seizure	A total of 2.2% developed with less than 1800 mg/day. A total of 0.6% developed with 1800–2400 and 2400–3600 mg/day.	NA	NA	NA	NA
Perugi et al. (1999) * [106]	1	NA	NA	NA	NA	NA	NA	NA
Dallocchio et al. (2000) * [107]	1	78; F	DM neuropathy	1600	NA	NA	NA	NA
Mellegers et al. (2001) * [108]	19	NA	NA	NA	NA	NA	NA	NA
Wilton et al. (2002) * [109]	NA	NA	NA	NA	NA	NA	NA	NA
Klein-Schwartz et al. (2003) [110]	1	42; NA	Intoxication	1200	NA	NA	NA	NA
Karagoz et al. (2013) [111]	3	71 (mean); 2F, 1M	Neuropathic pain	1200	NA	NA	CR	NA
Pal et al. (2014) * [112]	NA	NA	NA	NA	NA	NA	NA	NA
Kwon et al. (2019) [113]	NA	NA	NA	NA	NA	NA	NA	NA
Ghayur et al. (2021) [114]	1	57; F	Neuropathic pain	300	3 months	NA	NA	NA

Abbreviations: CR, complete recovery; CT, computed tomography; DM, diabetes mellitus; EEG, electroencephalogram; EMG, electromyography; ET, essential tremors; F, female; GBP, gabapentin; M, male; MCL, myoclonus; MD, movement disorder; MRI, magnetic resonance imaging; N, number of the patients reported; NA, not available/not applicable. ^a^ MD onset was defined as the time from the GBP start until the development of the abnormal movement. ^b^ MD recovery was defined as the time from the first management until the complete recovery of the motor symptom. * All types of study, except case reports, case series, and brief reports of cases.

**Table 3:** Clinical history and management of gabapentin-associated movement disorders.

Reference	CH and CM
Myoclonus	
Reeves et al. (1996) [19]	CH: She reported muscle twitches. These occurred once a minute and involved arm, leg, and neck muscles. The twitches were painless and were not of sufficient magnitude to cause the limb or head to move. After one week, the MCL disappeared, and GBP continued. After four weeks, the MCL reappeared. GBP was discontinued with complete recovery within four days.
Scheyer et al. (1996) [20]	CM: discontinue GBP and recovery from movement disorder.
Wilson et al. (1998) [21]	Refractory partial epilepsy (all the 50 patients in the study).
Asconape et al. (2000) [22]	Three patients were tapered off GBP, and the MCL completely resolved (two cases) or returned to baseline frequency (one case). In eight cases, in which GBP was continued at the same dose, six showed no change in the MCL, whereas two had a gradual spontaneous improvement, but not complete resolution. One patient had a decline in the frequency of MCL after an increase in dosage. Finally, one patient had a decrease in dosage but no change in frequency. The period during which patients had MCL ranged from 2 to 12 months.
Jacob et al. (2000) [23]	Severe postherpetic neuralgia that did not respond to amitryptiline or carbamazepine, and these were discontinued 1 month before admission. A week later, the drug was reintroduced at 300 mg/d without any recurrence of asterixis and she reported 50% pain relief.
Huppertz et al. (2001) [24]	In one individual MCL decreased with reduction of GBP. In the other three, MCL was unchanged with a GBP increase.
Scullin et al. (2003) [25]	Discontinuation of GBP.
Sechi et al. (2004) [26]	GBP-dose reduced.
Babiy et al. (2005) [27]	GBP was discontinued.
Bookwalter et al. (2005) [28]	GBP-dose reduced.
Zhang et al. (2005) [29]	GBP was discontinued.
Han et al. (2006) [31]	GBP was discontinued.
Holtkamp et al. (2006) [32]	The patient had a worsening of renal function. The MCL was observed. GBP was discontinued.
Striano et al. (2007) [33]	GBP was discontinued.
Cho et al. (2008) [34]	GBP was discontinued.
Ege et al. (2008) [35]	GBP was discontinued.
Pierce et al. (2008) [36]	The patient presented with hearing loss, MCL, and confusion. GBP levels were high. GBP was discontinued, and a hemodyalisis was performed.
Healy et al. (2009) [37]	The patient had MCL with GBP and pregabalin. But, MCL was not observed with carbamazepine or amitriptyline.
Koide et al. (2009) [38]	Discontinuation of GBP or clonazepam add-on resulted in cessation of MCL with no serious sequela.
Onuigbo et al. (2009) [39]	Following prompt drug discontinuation and continued daily hemodialysis or peritoneal dialysis respectively, both patients were discharged home, in normal clinical condition, after 3 days.
Chau et al. (2010) [40]	GBP was discontinued. After two days, MCL improved. After two months, EEG was normal.
Honma et al. (2010) [41]	GBP was discontinued.
Choe et al. (2011) [43]	After increasing hemodialysis to three times a week and discontinuing GBP, MCL spontaneously resolved.
Kampf et al. (2011) [44]	She had MCL with morphine and GBP.
Prieto-Pérez et al. (2011) [45]	GBP was discontinued.
Guddati et al. (2012) [46]	GBP level 6.3 mcg/mL, managed with continuous venovenous hemodiafiltration.
Torregrosa-Juan et al. (2012) [47]	GBP was discontinued, hemodialysis was performed. Encephalopathy improved progressively.
Kagnoff et al. (2013) [49]	The movement disorder was sudden multi-focal jerking limb movements that were worse with action, multiple metabolic abnormalities, and a history of high-dose GBP exposure.
Wahba et al. (2013) [50]	GBP was discontinued.
Kaufman et al. (2013) [52]	In one, GBP was mantained, and in the other, GBP was discontinued. Hemodialysis was performed in both cases.
Shea et al. (2014) [53]	Clonazepam was prescribed. GBP-dose was maintaned.
Clark et al. (2015) [54]	GBP-dose was reduced 100 mg 3×/d, but there was no improvement of MCL. GBP was discontinued, and within two days the MCL improved.
Guner et al. (2015) [55]	GBP was discontinued.
Ozmenoglu et al. (2015) [56]	GBP was discontinued.
Schnitzer et al. (2016) [57]	GBP was discontinued.
Ahmad et al. (2017) [58]	GBP-dose was reduced 100 mg 3×/d, and the symptoms completely improved.
Khan et al. (2017) [59]	GBP was discontinued.
Kim et al. (2017) [60]	GBP was discontinued.
Zheng et al. (2017) [61]	Patient was in use of GBP. She had an abdominal surgery, which led to the worsening of renal function. GBP toxicity was observed. GBP was discontinued. The abnormal movement improved.
Perez et al. (2018) [62]	Patient had worsening of renal function due to contrast-induced nephropathy leading to GBP toxicity. GBP was discontinued, and hemodialysis was performed.
Desai et al. (2019) [63]	One patient received lorazepam. All patient GBP was discontinued.
Hui et al. (2019) [64]	GBP was discontinued.
Medsafe et al. (2019) [65]	GBP was discontinued.
Yeddi et al. (2019) [66]	Hemodialysis was performed. No description about the GBP-dose is provided.
Latief et al. (2021) [67]	Hemodialysis was performed. No description about the GBP-dose is provided.
Parkinsonism	
Zadikoff et al. (2007) [68]	The patient was in use of valproic acid and GBP. Both antiepiletics can cause PKN.
Dyskinesia	
Millichap et al. (1996) [72]	Severe mental retardation and seizures. CM: Diphenhydramine 25 mg IV. Full recovery with GBP discontinuation.
Chudnow et al. (1997) [73]	CM: Both patients experienced resolution of abnormal movements on discontinuation of the therapy. One patient developed recurrent choreiform movements after drug rechallenge.
Millichap et al. (1997) [74]	CM: Case 1 also receiving valproic acid, intermittent choreoathetosis occurred for many weeks after GBP was discontinued. In case 2, receiving phenytoin, a GBP rechallenge caused recurrence of choreoathetosis in 7 days but to a lesser degree; the reduced severity of movements was related to a reduction in dosage of phenytoin.
Norton et al. (2001) [75]	GBP was discontinued.
Lai et al. (2007) [76]	The patient had spontaneous spinal epidural hematoma that caused neuropathic pain. GBP was started due to the neuropathic pain.
Raju et al. (2007) [77]	GBP was discontinued.
Lai et al. (2008) [78]	GBP was discontinued.
Shin et al. (2008) [79]	Patient was in use of GBP, clonazepam, oral iron supplements, citalopram, and haloperidol, when he had worsening of chorea.
Twardowschy et al. (2008) [80]	GBP was discontinued.
Zesiewicz et al. (2008) [81]	GBP was discontinued.
Attupurath et al. (2009) [82]	GBP was discontinued.
Bonakis et al. (2009) [83]	The patient had paroxysmal kinesigenic dyskinesia, which worsened with GBP therapy.
Erol et al. (2009) [84]	GBP was discontinued.
Souzdalnitski et al. (2014) [86]	GBP was discontinued. Chorea improved within 2 weeks. Orofacial DKN improved within months of GBP withdrawal.
VanHook et al. (2017) [87]	GBP was discontinued.
Rahil et al. (2018) [88]	GBP was discontinued.
Hampton et al. (2019) [89]	GBP was discontinued.
Akathisia	
Childers et al. (1997) [90]	GBP was discontinued, and the abnormal movements improved within 48 h.
Stuttering	
Nissani et al. (1997) [92]	CM–GBP was discontinued and stuttering was gone after 4 days of discontinuation.
Choi et al. (2004) [93]	GBP was discontinued.
Zeldin et al. (2019) [94]	GBP was discontinued.
Dystonia	
Reeves et al. (1996) [19]	One minute after the patient received 2 mg intravenous lorazepam, all abnormal movements ceased. GBP was discontinued, and the movements did not recur.
Bernal et al. (1999) [95]	GBP discontinuation. Abnormal movements in the upper right limb persisted.
Palomeras et al. (2000) [96]	The authors described that by reducing propranolol dose to 40 mg/day, the DTN disappeared immediately.
Pina et al. (2005) [97]	GBP was discontinued.
Allford et al. (2007) [98]	In the first surgery, she had myoclonic movements involving mainly the upper limbs. The episode persisted for 3 h and was not relieved by benztropine. In a second surgery, she had axial DTN. Therapy with long-acting benzodiazepines was ineffective.
Rohman et al. (2014) [100]	Treated with an intravenous dose of procyclidine 10 mg.
Myokymia	
Brown et al. (2021) [101]	GBP holiday was started. After restarting on a lower dose of GBP with multimodal pain control, the patient continued to improve with diminution of his MCL, tremor, and gait instability.

Abbreviations: CH, clinical history; CM, clinical management; DTN, dystonia; EEG, electroencephalogram; GBP, gabapentin; PKN, parkinsonism.

**Table 4:** Summary of gabapentin-associated movement disorder.

MD		MCL	DKN	DTN	AKT	Stutter	MKM	PKN	Others	General Data
Cases (%)		135 (66.17%)	22 (10.78%)	7 (3.43%)	3 (1.47%)	3 (1.47%)	1 (0.49%)	1 (0.49%)	32 (15.68%)	204 (100%)
Continent (%)	Africa	0 (0%)	0 (0%)	0 (0%)	0 (0%)	0 (0%)	0 (0%)	0 (0%)	0 (0%)	0 (0%)
	Australia	1 (0.74%)	0 (0%)	0 (0%)	0 (0%)	0 (0%)	0 (0%)	0 (0%)	0 (0%)	1 (0.49%)
	Asia	24 (17.77%)	4 (18.18%)	1 (14.28%)	0 (0%)	1 (33.33%)	0 (0%)	0 (0%)	0 (0%)	30 (14.70%)
	Europe	46 (34.07%)	4 (18.18%)	5 (71.42%)	0 (0%)	0 (0%)	0 (0%)	0 (0%)	26 (81.25%)	81 (39.13%)
	N. America	64 (47.40%)	13 (59.09%)	1 (14.28%)	3 (100%)	2 (66.66%)	1 (100%)	1 (100%)	6 (18.75%)	91 (44.60%)
	S. America	0 (0%)	1 (4.54%)	0 (0%)	0 (0%)	0 (0%)	0 (0%)	0 (0%)	0 (0%)	1 (0.49%)
Sex (%)	Female	42 (31.11%)	5 (22.72%)	2 (28.57%)	1 (33.33%)	1 (33.33%)	0 (0%)	1 (100%)		52 (25.49%)
	Male	42 (31.11%)	11 (50%)	4 (57.14%)	0 (0%)	2 (66.66%)	1 (100%)	0 (0%)		60 (29.41%)
	Unknown	51 (37.77%)	6 (27.27%)	1 (14.28%)	2 (66.66%)	0 (0%)	0 (0%)	0 (0%)		92 (45.09%)
Age (year)	Rg	23–89	73–83	10–72	76	58–66	69	61		10–89 (Md: 57)
	Mn	53.98	57.2	42.5	76	62	69	61		54.54 (SD: 17.79)
GBP-dose (Mn mg)		1277.75	1133.33	1516.66	1800	900	9600	NA		1324.66 (SD: 1117.66; Rg: 100–9600; Md: 1033)
MD onset	Range	1 h–4 years	2 days–1 years	1 day–16 months	1 week	2 days	NA	NA		1 h–4 years
	Mean	4.01 weeks	2.51 weeks	2.80 weeks	1 week	2 days	NA	NA		4.58 weeks (Sd: 8.08 Md: 1)
MD recovery	Range	1 day–12 months	1 day–months	1 day–weeks	2 days	4 days–weeks	3 days	NA		1 day–12 months
	Mean	3.66 days	7.85 days	9 days	2 days	4 days	3 days	NA		4.17 days (Sd: 4.87 Md: 2.5)
Follow-up—% CR (number of reports)		79.24% (42/53)	88.23% (15/17)	83.33% (5/6)	NA (0/0)	100% (3/3)	100% (1/1)	NA		82.5% (66/80)

Abbreviations: AKT: akathisia; CR: complete recovery; DKN: dyskinesia; DTN: dystonia; MCL: myoclonus; MD: movement disorder; Md: median; MKM, myokymia; Mn: mean; NA: not available/not applicable; PKN: parkinsonism; Rg: range (minimum-maximum); SD: standard deviation. In the “Others” subgroup are cases not specified about the movement disorder such as extrapyramidal symptoms.

**Table 5:** Incidence of some abnormal movements associated with GBP in the literature.

MD	Incidence	N Cases	Population	Note	Reference
MCL	1.09%	8	729	Assessment of GBP dosage and risk of toxicity in patients with chronic kidney disease.	Zand et al. [42]
MCL	1.85%	3	162	NA	Koide et al. [38]
MCL	2.50%	1	40	Cross-over, open-label trial of the effects of GBP on peripheral neuropathy.	Atalay et al. [48]
ATX	2.60%	58	2216	Open-label multicenter study, which investigates how many patients become seizure free after GBP use.	McLean et al. [105]
ATX	3.60%	4	110	Open-label multicenter study, which investigates how many patients become seizure free after GBP use.	Mayer et al. [104]
MCL	4%	2	50	Efficacy of high-dose GBP in refractory partial epilepsy.	Wilson et al. [21]
ATX	4%	1	25	Open-label pilot study on GBP vs. amitriptyline in diabetic neuropathy.	Dallochio et al. [107]
MCL	4.22%	3	71	Patients with end-stage renal disease.	Zhang et al. [29]
ATX	4.76%	1	21	Adjunctive GBP therapy efficacy in bipolar disorder type I.	Perugi et al. [106]
Tremor	4.87%	4	82	Efficacy and safety of GBP administered as monotherapy in refractory complex partial or secondarily generalized seizures.	Bergey et al. [102]
Choreoathetosis	7.14%	2	28	NA	Millichap et al. [74]
ATX	7.42%	19	256	NA	Mellegers et al. [108]
ATX	12.19%	10	82	Efficacy and safety of GBP administered as monotherapy in refractory complex partial or secondarily generalized seizures.	Bergey et al. [102]
MCL	12.50%	13	104	NA	Asconape et al. [22]
ATX	14.16%	34	240	Safety of GBP as add-on therapy in patients with refractory partial seizures.	U.S. Gabapentin Study Group [6]
Tremor	15%	36	240	Safety of GBP as add-on therapy in patients with refractory partial seizures.	U.S. Gabapentin Study Group [6]
Asterix	15.58%	12	77	NA	Kim et al. [60]
MCL	21.05%	4	19	NA	Huppertz et al. [24]

Abbreviations: ATX, ataxia; MCL, myoclonus; MD, movement disorder; NA, not available/not applicable.

**Table 6:** Gabapentin and pregabalin-associated movement disorder.

MD		Gabapentin General Data	Pregabalin General Data ^a^
Cases (%)		204 (100%)	305 (100%)
Movement disorders	Akathisia	3 (1.47%)	1 (0.32%)
	Ataxia	NA	184 (60.32%)
	Dyskinesia	22 (10.78%)	1 (0.32%)
	Dystonia	7 (3.43%)	1 (0.32%)
	Myoclonus	135 (66.17%)	39 (12.78%)
	Myokymia	1 (0.49%)	0 (0%)
	Parkinsonism	1 (0.49%)	8 (2.62%)
	Restless legs syndrome	0 (0%)	1 (0.32%)
	Stuttering	3 (1.47%)	0 (0%)
	Tremors	NA	61 (20%)
	Others	32 (15.68%)	9 (2.95%)
Continent (%)	Africa	0 (0%)	0 (0%)
	Australia	1 (0.49%)	0 (0%)
	Asia	30 (14.70%)	40 (13.11%)
	Europe	81 (39.13%)	68 (22.29%)
	N. America	91 (44.60%)	196 (64.26%)
	S. America	1 (0.49%)	1 (0.32%)
Sex (%)	Female	52 (25.49%)	21 (6.88%)
	Male	60 (29.41%)	25 (8.19%)
	Unknown	92 (45.09%)	259 (84.91%)
Age (year)	Rg	10–89 (Md: 57)	23–94 (Md: 66.5)
	Mn	54.54 (SD: 17.79)	62.89 (SD: 18.12)
Dose (Mn mg)		1324.66 (SD: 1117.66; Rg: 100–9600; Md: 1033)	238 (SD: 136.95; Rg: 50–600; Md: 150)
MD onset	Range	1 h–4 years	1 day–9 months
	Mean	4.58 weeks (Sd: 8.08; Md: 1)	9.48 days (Sd: 16.78; Md: 3)
MD recovery	Range	1 day–12 months	1 day–6 months
	Mean	4.17 days (Sd: 4.87; Md: 2.5)	12.17 days (Sd: 22.13; Md: 2.5)
Follow-up—% CR (number of reports)		82.5% (66/80)	100% (18/18)

Abbreviations: CR: complete recovery; MD: movement disorder; Md: median; Mn: mean; NA: not available/not applicable; Rg: range (minimum–maximum); SD: standard deviation. In the ‘‘Others’’ subgroup are cases not specified about the movement disorder, such as extrapyramidal symptoms. ^a^ Data extracted from the reference Rissardo et al. (2020) [140].

## Data Availability

Not applicable.
